# Participation in modified sports programs: a longitudinal study of children’s transition to club sport competition

**DOI:** 10.1186/s12889-015-2012-y

**Published:** 2015-07-14

**Authors:** Rochelle M. Eime, Meghan M. Casey, Jack T. Harvey, Melanie J. Charity, Janet A. Young, Warren R. Payne

**Affiliations:** School of Health Sciences and Psychology, Federation University Australia, Ballarat, Australia; Institute of Sport, Exercise and Active Living (ISEAL), Victoria University, Melbourne, Australia

## Abstract

**Background:**

Many children are not physically active enough for a health benefit. One avenue of physical activity is modified sport programs, designed as an introduction to sport for young children. This longitudinal study identified trends in participation among children aged 4–12 years. Outcomes included continuation in the modified sports program, withdrawal from the program or transition to club sport competition.

**Methods:**

De-identified data on participant membership registrations in three popular sports in the Australian state of Victoria were obtained from each sport’s state governing body over a 4-year period (2009–2012 for Sport A and 2010–2013 for Sports B and C). From the membership registrations, those who were enrolled in a modified sports program in the first year were tracked over the subsequent three years and classified as one of: transition (member transitioned from a modified sport program to a club competition); continue (member continued participation in a modified sport program; or withdraw (member discontinued a modified program and did not transition to club competition).

**Results:**

Many modified sports participants were very young, especially males aged 4–6 years. More children withdrew from their modified sport program rather than transitioning. There were age differences between when boys and girls started, withdrew and transitioned from the modified sports programs.

**Conclusions:**

If we can retain children in sport it is likely to be beneficial for their health. This study highlights considerations for the development and implementation of sport policies and programming to ensure lifelong participation is encouraged for both males and females.

## Background

Many children and adolescents do not participate in sufficient levels of physical activity for health benefits [1]. For children (5–17 years) it is recommended that they undertake moderate and/or vigorous activities for at least 60 min per day [2]. However, international reports indicate that up to 80 % of 13–15 year olds do not meet this guideline [1]. In Australia, only one in five children aged 13–15 years of age (19 %) have been reported to do the recommended 60 min per day across all seven days, although based on self-report almost half (48 %) met the recommendation on at least five of the previous seven days [3]. Many children and youth are engaged in high levels of sedentary time which impact negatively on their health [4]. The physical activity guidelines also highlight the fact that any level of physical activity is better than none at all [5].

Organised sport is one type of leisure-time physical activity that can help children achieve physical activity recommendations. An estimated 60 % of Australian children (aged 5–14 years) participate in at least one sport organised by a community sports club outside of school hours [6]. Participation in sport for children can be associated with improved psychosocial health above and beyond improvements attributed to participation in physical activity [7]. Specifically, team sport has been shown to have improved health outcomes for children, due to its social nature of participation, compared to individual physical activity pursuits [7].

In terms of the opportunities for organised sport participation outside of school hours, young children can participate in modified sports programs as a pathway to club sport competition. In Australia modified sport programs are offered for primary school-aged children (generally 4–12 years), although the upper age limit varies from program to program. Modified sports include, but are not limited to: ‘Hot Shots’ in Tennis [8] ‘AusKick’ in Australian Rules Football [9] ‘MiniRoos’ in Soccer or Football [10] and ‘NetSetGo’ in Netball [11].

Modified sports programs are offered to engage children in play activities designed, among other things, to develop fundamental motor skills and sport-specific skills for future participation [12]. Essentially, the sport is modified to match the developmental capabilities of children by adapting games and activities through changes to the rules, equipment, and/or physical space to encourage inclusion and maximise participation. The fundamental focus of modified sport programs is on learning and development, including developmentally appropriate competition but not on competition per se.

The number of children participating in modified sports has increased in recent times [13], which is likely to have been influenced by deliberate strategies employed by State and Commonwealth governments and health promotion organisations to encourage sport participation in general, and also by sport governing bodies offering modified sport to encourage more young people to participate in organised sport [14, 15].

To our knowledge there have been no studies that have investigated the participation patterns of children in modified sports programs, including children’s transition from modified sport programs to club sport competition or withdrawal from sport. The purpose of this study was to identify longitudinal trends in modified sport participation among children in three popular sports in Australia, including 1) transition over a 4-year period from modified sports program to club sport competition in the same sport or withdrawal from that sport, and 2) sex differences in transition and withdrawal patterns.

## Methods

The three sports studied were ranked within the top 10 Australian organised sports for participation by children aged 5–12 years [6]. De-identified membership registration data for these sports in the state of Victoria were obtained from the respective state sport governing bodies, generically referred to as state sporting associations. A condition of provision of these data for this study was that the particular sports would not be identified in research outputs. Data from each sport encompassed four annual registration cycles, (2009–2012 for sport A and 2010–2013 for sports B and C). A participant was defined as a registered member of a modified sport program centre and/or club affiliated with the state sporting association. In all but the youngest age groups, a member may have been a member previously, at any time prior to 2009 (sport A) or 2010 (sports B and C). Ethics approval was granted by the University Human Research Ethics Committee for secondary analysis of the de-identified membership data without explicit consent of the participants or their parents/caregivers.

State sporting associations provided us with data for each registered member in each of the four years of the study, including a unique identifier, sex, date of birth, and program. We identified 4–12 year-old participants in modified sport programs in Year 1 (2009, sport A and 2010, sports B and C), and using the unique identifiers tracked them over the 4-year period. Each member was classified as one of:

- Transition – member transitioned from the modified sport program to a club sport competition within the same sport during the 4-year period.
- Continue – member continued participation in the modified sport program in the same sport throughout the 4-year period.
- Withdraw – member discontinued participation in the modified program of the particular sport during the 4-year period, and did not transition to a club sport competition in that sport.

All data were integrated and data for the three sports were analysed collectively in order to produce broad-based participation profiles while maintaining confidentiality of membership data for individual sports. The age breakdowns presented are based on the age of each member in Year 1.

Because of anonymity provisions, it was not possible to identify participation of a particular individual in more than one sport in Year 1. An individual could engage in more than one sport and if so was counted separately in each sport, with the result that counts of participants are to some extent inflated or ‘weighted’ by individuals’ overall levels of participation. For the same reason, we were unable to identify or quantify transition from one sport into another.

The outcome variables were the proportions of participants in each age/sex cohort whose progression over the 4-year period fell into each of the three categories above (transition, continue, withdraw). Because data were collected from the whole population of members of each modified sport in the base year, rather than a sample, statistical inference was not required. Analyses were conducted using Excel and SPSS Version 21.

## Results

**Fig. 1:**
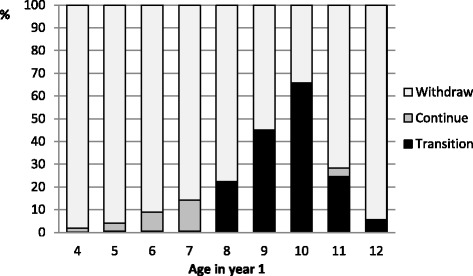
Modified sport program outcomes for females: by age in year 1

**Fig. 2:**
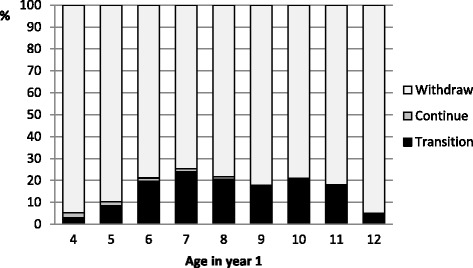
Modified sport program outcomes for males: by age in year 1

**Fig. 3:**
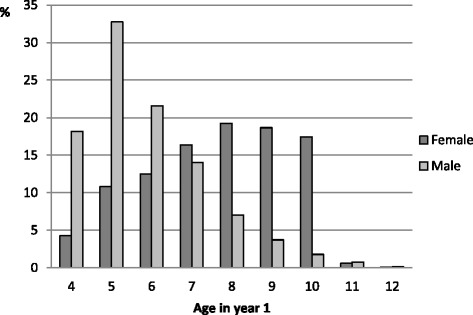
Modified sport participation: age profiles by sex

Table 1 shows that a total of 209,336 children (female 36.4 % and male 63.6 %) participated in one of three modified sports programs in the base year. Numbers of participants at year 1 for each age group (4–12 years) are shown in Table 2 and Fig. 1, and the percentage of each age group who were classified as transition, continue, and withdraw are shown in Figs. 2 and 3. The majority of female children were aged 8–10 years (*n* = 42,159; 55.4 %) compared with males who were younger, aged 4–6 years (*n* = 96,572; 72.5 %).

**Table 1:** Number participants in a modified sport program in year 1: by sport and sex

		Female		Male		Total
	Year 1	n	%	n	%	
Sport A	2009	3,442	10.7	28,872	89.3	32,314
Sport B	2010	11,075	9.7	103,196	90.3	114,271
Sport C	2010	61,633	98.2	1,118	1.8	62,751
Total		76,150	36.4	133,186	63.6	209,336

**Table 2:** Age profiles of female and male participants in year 1

Age	4	5	6	7	8	9	10	11	12	Total
Females										
N	3,249	8,233	9,535	12,472	14,664	14,206	13,289	447	55	76,150
%	4.3	10.8	12.5	16.4	19.3	18.7	17.5	0.6	0.1	100
Males										
N	24,210	43,627	28,735	18,709	9,400	4,948	2,373	1,025	159	133,186
%	18.2	32.8	21.6	14.0	7.1	3.7	1.8	0.8	0.1	100

Across all age groups, fewer than 25 % of females (*n* = 18,652; 24.5 %) and fewer than 14 % of males (*n* = 18,058; 13.6 %) transitioned from a modified sports program to a club sport competition within the 4-year period. Very few children continued in a modified sports program for the whole 4-year period (females: *n* = 2,934; 3.9 % and males: *n* = 2,149; 1.6 %).

Figures 2 and 3 also illustrate that, across all age groups, the majority of children participating in a modified sports program withdrew before transitioning to a club sport competition in the same sport (females: *n* = 54,564; 71.7 % and males: *n* = 112,979; 84.8 %). Further analysis not shown in the figures revealed that of those children who withdrew from a modified sport program, two-thirds withdrew immediately after the base year/season (*n* = 113,086; 67.4 %), although it does not necessarily follow that they only participated for one year – for all but the 4-year-olds, they may have already been participating prior to the base year of the study.

The proportions displayed in Figs. 2 and 3 must be interpreted with care, in conjunction with the numbers of participants displayed in Table 2. For example, the age from which the highest proportion of females transitioned from a modified program to a club sport competition during the 4-year period was 10 years (*n* = 8,742; 65.8 %).

The peak age for transition for males was much younger than females at 7 years (*n* = 4,496; 24.0 %), although, male transition was fairly consistent across the age range 6–11 years. However, participation numbers for males were much higher in the youngest age groups - 4 (*n* = 24,210), 5 (*n* = 43,627) and 6 years (*n* = 28,735), which together represented 72.5 % of males aged 4–12 years, in contrast to 30.2 % of females.

## Discussion

This study uniquely describes, using a large sport participation cohort, the participation patterns of young children in three sports and their withdrawal or transition from modified sports programs into club sport competitions. It is clear that most children do not transition from the modified sport program into club sport competitions in the same sport. However, there are a number of differences across age and sex.

This study showed that a high proportion of modified sport participants were young, especially males (4–6 years). Most did not transition to the affiliated club sport competition within a 4-year period; indeed, two-thirds of those who withdrew from modified sports programs did so immediately after the base year of the study, from which it is conjectured that many had only a single year/season of participation.

It is reported that young children tend to ‘sample’ sports during the ‘sampling years’ when they are given a range of different sports activities to ‘try out’ and through which they can develop fundamental motor skills [16]. As children age they tend to specialise (‘specialising years’) and focus on just one or two sports after which they either move to the ‘investment’ or ‘recreational’ years [16]. In light of sampling behaviour, high withdrawal rates from modified sports programs are perhaps to be expected, and as such these results may not necessarily indicate a problem. From a public health perspective, if early registration in a modified sport program fosters prolonged participation in any form of physical activity, it does not matter what ultimate form that takes. Of course, sports organisations may have a narrower self-interested focus. Sampling (also known as early diversification) has been linked to long-term sport involvement, and it is recommended that sport programs encourage early involvement in different sports and contexts [17]. Further, it is posited that children who sample a variety of sports are exposed to unique socialisation experiences that shape personal development and social capital outcomes including intrapersonal skills, pro-social behaviour, healthy identity and diverse peer groups [17].

Regardless of whether it be through sampling or continuing in the one sport, there are considerable benefits if children can play sport continuously, instead of dropping out altogether. Recent literature suggests that children (aged 6–9) who participated continuously (over 3 years) in a club environment had better coordination levels than those who were discontinuous or did not participate at all [18]. Furthermore, the basic level of motor coordination and the amount of club sport participation significantly predicted sport participation two years later [18]. These outcomes have implications for elite pathways (investment years), as well as health-related benefits of sport participation (recreational years) in childhood and throughout the lifespan [18].

Whilst this study represents only three (albeit popular) sports, for these sports, very young males (ages 4–6) represented a much higher proportion of all male participants (72.5 %) than was the case for females (30.2 %). This may be linked to consistent reports of lower sport participation rates among females [19–21]. For instance, there is evidence that parents provide sons with more opportunities for sport participation than daughters [22]. Verbal encouragement, support, and active participation from family members has been reported to help girls be physically active, along with strategies that focus on peers, the school and community to shape positive perceptions and attitudes towards girls’ participation in physical activities [23]. It is important that girls are provided with opportunities to develop their motor coordination early, as the critical developmental period for motor development is before the age of five or six [24].

The present study also found that as a result of males being engaged in modified sport at earlier ages, the peak age of transition from a modified sport program to club sport competition was lower among males (7 years of age in the base year) than females (10 years of age). This may be because males perceive that they have become competent in the modified sport and are ready for competition, or perhaps they become ‘bored’ with the modified sport program and seek new challenges. Whilst transition to club sport competition does not necessarily mean that these children are ‘specialising’ in the sport, it is likely to involve/require higher levels of challenge and engagement than a modified sports program. Many club sport competitions involve training as well as competition, additional costs associated with memberships and uniforms, and additional costs and time associated with travel to games. As a result of these increased demands, it is likely that these children may be starting to move from sampling to specialising at early ages. It has been suggested that specialisation should not occur before the age of 13 in sports where peak performance is reached in adulthood [17], particularly as sampling provides opportunities to develop physical competencies and perceptions of competence which lead to motivation for continued participation [25]. Interestingly, the peak rate of sport participation for both males and females in Australia has been reported to occur at around 11 to 13 years of age. Eime et al. [26] reported peak sport participation in five popular Australian sports at 10 and 11 years for boys and girls respectively. Other researchers have reported slightly higher sport participation peaks at ages 12–13 years in a national sample, and a decline of 50 % by age 16 [19].

This study extends the findings of others who have reported that boys are more active than girls [1], and highlights the fact that, at least in the three sports examined, boys tend to participate in organised sport much earlier than girls, and as result seek new challenges much earlier. However, it is possible that the available challenges may not be providing an environment that promotes lifelong involvement in sport for both boys and girls [27] as this study found that most participants of both sexes tend to withdraw from a particular modified sport program rather than transitioning into club competition in the same sport.

Modified sport programs are designed for children aged 4–12, however this current study has identified that many children participating in these programs are in the lowest part of the age-range (4–5 years). Given the developmental capacity required to play competitive club sport, perhaps very young children are getting bored after 1–2 years, but may not be old enough to play club competition, so they either drop-out of sport altogether or sample to another sport. Therefore for continued participation in a given sport, an intermediate program in the sport participation pathway may be required. An example is Tennis Hot Shots Leagues which is aligned to the modified program and provides children with a chance to practice their skills in a modified competition structure, with modified balls and scoring [28].

The drive for children to focus on higher levels of engagement in sport at earlier ages may also influence their perceptions of competence. Children’s perceptions of competence in late childhood (ages 8–12) is largely the result of comparison with their peers [17]. Comparisons of motor skill proficiency and physical activity performance are commonly reported as factors influencing non-sport participation, and are often influenced by peer teasing [23, 29, 30]. One study reported that adolescent girls perceived sports clubs as being exclusively for skilled participants, which regulated their non-participation in this setting (Casey M, Mooney A, Smith J et al. Power, regulation and physically active identities: The experiences of rural and regional living adolescent girls. Gender and Education 2014, Under Review). Others have also reported that the dominance of particular sports activities, particularly in regional and rural communities, can significantly limit the meanings constructed about being physically active, whereby some sporting activities are considered the privilege of the skilled, attractive and popular social elite [30]. If children are transitioning to club competition at early ages (7 for males and 10 for females) they may be participating with children who are older and who have greater physical competency, and consequently impacting their perceptions of competence and subsequent motivation for continued participation. Further, the modified sports programs investigated in this study are often delivered unisex, and therefore do not take into account differences between sexes. This study showed that boys and girls tend to start and withdraw or transition from the modified sports programs at different ages, whilst other studies have reported sex differences in the acquisition of motor skills for children [31]. Therefore, the same program may not be suitable for both boys and girls of similar ages, as their level of competence and mastery of fundamental motor skills are likely to differ.

This study had a number of limitations. The findings of this study were limited to only three Australian sports, albeit popular ones, and cannot necessarily be generalised to sports in general. Two of the three sports were dominated by males and one by females, although this imbalance did not limit our ability to identify sex differences in the patterns of participation. Data limitations and anonymity provisions precluded us from identifying or quantifying the proportions of participants in multiple sports in the base year or those who transferred into a different sport in later years. According to a recent national survey [32], 28.6 % of Australian children aged 5–14 years (34.8 % of boys and 22.2 % of girls) reported playing more than one sport in the 12 months prior to interview; however this included all sports, compared with only three sports in the present study, and much of it would be attributable to participation in different sports at different times of the year. Further, we assumed that non-registration in a later year implied withdrawal from the particular sport, whereas a proportion of assumed withdrawals may have migrated interstate but maintained their participation in the sport; however, considering that outward migration in 2010–11 amounted to 1.1 % of the Victorian population [33, 34], the effect on our conclusions is minimal. A strength of the study was its inclusion of the total population of participants in three representative modified sports in a large sub-national region. Future research should examine a range of modified sports programs and club sport competitions to better understand participation and withdrawal trends and to understand the socio-ecological factors influencing children’s participation, transition and withdrawal from modified sports programs.

## Conclusions

The findings of this study are consistent with theoretical notions of sampling and specialisation years [16]. There were fewer modified sports participants with increasing age, and very few children transitioned to club competitions in the same sport within a 4-year period. There were also differences between boys and girls with regard to when they started and when they transitioned or withdrew from the modified sports programs. This study highlights considerations for the development and implementation of sport policies and programming, whereby the same program may not be suitable for both boys and girls, nor across the age spectrum of 4–12 year. Further, the results of this study suggest that there is a need for better links between modified programs and club sport competition programs if continuity of participation in a particular sport is to be maintained as children age. The inclusion of an intermediate program within the sport participation pathway, between modified sport and club sport competitions, may assist continuation of participation in a given sport.
